# Dietary Surveys and Nutritional Epidemiology

**DOI:** 10.3390/nu17020263

**Published:** 2025-01-12

**Authors:** Margarida Liz Martins, Sandra Abreu

**Affiliations:** 1 Coimbra Health School, Polytechnic University of Coimbra, 3045-093 Coimbra, Portugal; 2 H&TRC—Health & Technology Research Center, Coimbra Health School, Polytechnic University of Coimbra, 3045-093 Coimbra, Portugal; 3 Sports and Physical Activity Research Center, University of Coimbra, 3040-248 Coimbra, Portugal; 4 Research Centre for Anthropology and Health, University of Coimbra, 3000-456 Coimbra, Portugal; 5 School of Life Sciences and Environment, University of Trás-os-Montes and Alto Douro (UTAD), 5000-801 Vila Real, Portugal; sandraabreu@utad.pt; 6 Research Centre in Physical Activity, Health, and Leisure (CIAFEL), Faculty of Sport, University of Porto, 4200-450 Porto, Portugal; 7 Laboratory for Integrative and Translational Research in Population Health, 4050-600 Porto, Portugal

As the prevalence of lifestyle-related diseases continues to increase globally [1,2] and the social determinants of health increasingly influence dietary patterns, there is a pressing demand for robust methodologies to accurately capture dietary intake and assess its impact on health outcomes across diverse populations and contexts. Nutritional epidemiology bridges this gap by leveraging dietary surveys and data to elucidate the complex relationship between diet and health, enabling more effective public health strategies and policies [3].

This Special Issue of *Nutrients* compiles significant research contributions that broaden the scope, accuracy, and applicability of dietary assessments. The studies featured various cultures, age groups, and specific dietary components, utilizing both established and innovative methodologies to evaluate food and nutrient intake. Importantly, these articles highlight the crucial need for surveys that can adjust to the socio-cultural context of populations while upholding the need for reliability and validity in dietary assessments.

Within this Issue, there are advancements in the design and validation of culturally specific dietary assessment tools, enhancements in digital and web-based dietary recall systems, and sophisticated statistical approaches to optimize food frequency questionnaires (FFQ). The insights gained from these studies offer guidance on tailoring dietary surveys to specific populations, recommended nutrient intake, and the incorporation of genetic and behavioral data to personalize dietary interventions. Furthermore, by exploring particular dietary components, food and beverage consumption patterns, and their impact on health, this Issue stresses the significance of understanding all dietary behaviors within the broader context of nutritional epidemiology.

The research presented in this Special Issue not only enhances the methodological rigor of dietary assessment tools, it also offers practical insights into how diet–disease relationships may be influenced by factors such as meal timing, cultural dietary habits, genetic polymorphisms, and reporting biases. Collectively, these contributions reflect the dynamic relationship between diet and health and emphasize the role of accurate, culturally relevant dietary surveys in promoting a healthier global population.

Murakami et al. (Contribution 1) significantly contribute to the Japanese context by validating both the web and paper versions of the Meal-Based Diet History Questionnaire (MDHQ) against traditional weighed dietary records. Their evaluation of the questionnaire’s accuracy in estimating food intake across different meal types and food groups establishes a strong framework for high-precision dietary assessment in Japanese adults. This study is complemented by another study (Contribution 2) by the same team that expands the questionnaire’s validation to a broader demographic, capturing the complexity of nutrient intake across different age groups and genders. Their findings show an acceptable level of relative validity for estimating nutrient intake compared to a 4-day weighed dietary record, although there are limitations in its ability to accurately estimate nutrient intakes at the individual level. These studies collectively support the use of the MDHQ in large-scale epidemiological and intervention research in Japan for assessing dietary intake patterns. These findings are in line with research by Kelly et al. (Contribution 3) on the UK Biobank’s Individual Fatty Acid Dataset, where they analyze fatty acid consumption patterns in a large cohort. Together, these studies demonstrate how robust datasets and culturally adapted tools can provide insights into population-wide dietary patterns and nutrient intake, with significant implications for public health policy and intervention strategies.

Moving from nutrient-specific to temporal dietary patterns, Lin et al. (Contribution 4) present a novel approach to uncover data-driven temporal dietary patterns based on meal timing and energy cut-offs. This study provides a unique temporal perspective, intersecting with Murakami’s research (Contribution 1) by adding a nuanced dimension that accounts for not only the composition of dietary intake but also its timing, revealing opportunities to explore diet–disease relationships and personalize dietary interventions based on eating habits. Alruwaitaa et al. (Contribution 5) extend this concept of cultural adaptation to a different region by validating the Arabic version of the Adult Eating Behavior Questionnaire. This research highlights the importance of linguistic and cultural adaptations in dietary assessments, emphasizing the need for precision across varied populations for more meaningful public health applications.

To enhance their practical usability, Laramée et al. (Contribution 6) compare the user experience of two web-based dietary recalls, R24W and ASA24-Canada-2018, among French-speaking adults in Québec. Their findings underscore the importance of accessible and user-friendly tools tailored to regional language and digital literacy requirements. This need for culturally and linguistically responsive tools is also evident in Igarashi et al.’s study (Contribution 7), which investigates dietary behaviors in the context of genetic predispositions, particularly the ALDH2 rs671 polymorphism. This research highlights the intricate interplay between genetic factors and dietary preferences, suggesting that more personalized dietary approaches may be essential in specific population subgroups, particularly in Asian populations, in which this polymorphism is highly prevalent.

Consideration of factors associated with self-reported dietary assessment also emerges as a key theme in Conway et al.’s study (Contribution 8) of cancer survivors. Their findings underscore the critical need for tailored assessment methods in vulnerable populations, such as older individuals and those not educated to degree level. Silva et al.’s research (Contribution 9) focuses on improving iodine intake estimates for pregnant women by incorporating salt and seasoning as covariates in the statistical methods. Both studies reinforce the message that accurate dietary assessment requires consideration of the unique characteristics of specific population groups.

The FFQ is widely used in epidemiological studies, and optimizing it is crucial for improving the assessment of habitual dietary intake. Rostgaard-Hansen et al. (Contribution 10) validated a web-based FFQ within Denmark’s Diet, Cancer, and Health Study, contributing to the growing field of digital dietary assessment tools. Blaurock et al. (Contribution 11) pushed the methodological boundaries further by using mixed-integer linear programming to optimize food frequency lists, demonstrating how computational advances can increase the accuracy of the data while reducing the participant burden. Additionally, Guzmán et al. (Contribution 12) adapted and validated a short FFQ for the quantification of dietary fiber intake in Chilean adults, facilitating the estimation of dietary fiber and the classification of individuals based on their habitual dietary fiber intake. A food group-based FFQ was also validated to assess the consumption of sugar-sweetened beverages and added sugar among adolescents (Contribution 13). The questionnaire demonstrated good validity and reproducibility, establishing it as a reliable tool for epidemiological research on sugary beverage consumption among adolescents.

Finally, we can observe the broader applications of dietary survey techniques in Bi et al.’s study (Contribution 14), which provided critical insights into beverage consumption patterns among U.S. adolescents and adults using a new 24 h Beverage Recall Survey. Moreover, in the scoping review by Krasnovsky et al. (Contribution 15), the relationship between nutritional biomarkers and food security is examined. The study emphasizes the complexity of using biomarkers associated with food security due to variations influenced by individual health, genetics, and environmental factors. This review underscores the need for comprehensive strategies to integrate biomarkers into food security assessments for more targeted interventions.

This Special Issue demonstrates that dietary surveys have evolved considerably, yet the field continues to innovate to capture the nuances of diet in diverse populations, as well as the interplay of temporal, cultural, genetic, and methodological factors that influence nutritional epidemiology. With these advancements, we are better equipped to understand diet–disease relationships and contribute to tailored, culturally sensitive, and actionable public health strategies worldwide.

## References

[1] GBD 2021 Forecasting Collaborators (2024). Burden of disease scenarios for 204 countries and territories, 2022–2050: A forecasting analysis for the Global Burden of Disease Study 2021. Lancet.

[2] GBD 2021 Causes of Death Collaborators (2024). Global burden of 288 causes of death and life expectancy decomposition in 204 countries and territories and 811 subnational locations, 1990–2021: A systematic analysis for the Global Burden of Disease Study 2021. Lancet.

[3] Satija A., Yu E., Willett W.C., Hu F.B. (2015). Understanding nutritional epidemiology and its role in policy. Adv. Nutr..

